# Comparison of Hydroxypropyl Methylcellulose and Alginate Gel Films with Meloxicam as Fast Orodispersible Drug Delivery

**DOI:** 10.3390/gels9090687

**Published:** 2023-08-25

**Authors:** Barbara Jadach, Martyna Misek, Jan Ferlak

**Affiliations:** Chair and Department of Pharmaceutical Technology, Poznan University of Medical Sciences, 60-780 Poznan, Poland; martyna.misek165@gmail.com (M.M.); janferlak@poczta.onet.pl (J.F.)

**Keywords:** polymer gel film, orodispersible gel film, meloxicam, dissolution, fast disintegration

## Abstract

The aim of the study was the preparation and comparison of two types of orodispersible gel films (ODF) by the solvent casting method. Natural polymers: sodium alginate (ALG) or hydroxypropyl methylcellulose (HPMC) were used as the gel film formers, and Kollidon or microcrystalline cellulose was used as the disintegrant. Meloxicam (MLX), the drug used to treat rheumatic diseases for children and adults, was proposed as the active pharmaceutical ingredient (API). The influence of the polymer and disintegrant on the properties of ODF was investigated. The evaluation of prepared gel films was based on appearance description, mass uniformity measurement, disintegration time, API content, film wettability, and water content. Also, the dissolution test was prepared in a basket apparatus using artificial salvia (pH = 6.8) as the medium. The obtained API release profiles were analyzed for the similarity factors (f_2_) with the DDSolver software. The results showed that independently of the polymer or disintegrant, using the solvent casting method, gel films have a similar appearance and active substance content close to the theoretical value and water content of less than 10%. Only the type of polymer influences the release profiles of MLX. However, the disintegration time was longer than 30 s, which makes the films non-fast-dissolving drug delivery systems. This means that for the ODF system, further evaluation is required, and some changes in the composition of the film have to be done.

## 1. Introduction

Polymer gel films, also known as polymer films, orally disintegrating membranes or films, lamellae, or ODF (Oro-dispersible films) are oral drug forms. They can be found as oblong, round or other shapes with an area of 2 to 8 cm^2^, are usually flat with a thickness ranging from 12 to 100 µm, and consist of one or more polymer gel layers [1,2,3,4,5,6,7]. As many as 37% of surveyed patients have difficulty swallowing tablets and capsules. The advantage of using drugs in the form of orodispersible polymer films is that there is no need to swallow them, which works well for children, the elderly, or patients with dysphagia [4,5,6]. They are placed in the oral cavity, where they should disintegrate in approximately 30 s and release the active substance [1]. After the oral membrane breaks down, the released API is swallowed by the patient, and then the substance is absorbed into the bloodstream. Taking the drug in this form does not require chewing or drinking water; the gel film is placed on the tongue, and its disintegration occurs after contact with saliva in the oral cavity [3,4,5,7]. According to the FDA (Food and Drug Agency) [8], 4% of patients cease taking medications in the form of tablets due to problems with swallowing. ODFs are a form perfectly suitable for elderly patients, children, people who have problems or fears related to swallowing, patients after surgery of the upper sections of the gastrointestinal tract, patients who do not cooperate, or patients in places without access to drinking water. Compared to orally-disintegrating tablets, polymer films are flexible and do not crumble, facilitating their dosing and taking up less space [9,10,11]. When using film strips, there is no risk of taking the wrong dose, as in the case of dispensing liquid forms of drugs, because they contain a precisely measured amount of the drug in one portion. There is no risk of choking when using ODFs. The limitation that can be faced when choosing a drug in the form of polymer gel membranes is their capacity. Due to their size, the lamellas can contain small doses of medicinal substances, which is why they are good for strong APIs [12,13,14,15]. Due to the release of the drug in the mouth, as well as the fact that the taste of API is often not pleasant, there is a need to use flavor enhancers, which additionally reduce the capacity of films for medicinal substances [3,6]. Another limitation is the lack of stability of the gel films in a humid environment. ODFs are also not used for substances that are unstable in the pH of the oral cavity or substances that irritate the area of the oral cavity and tongue [5]. The lamellas consist primarily of API, a properly selected polymer, disintegrants, plasticizers, and substances that improve the appearance and taste [6]. ODFs are an attractive and innovative drug form that differ from traditional and popular tablets and other known formulations [6,11,14]. They usually contain strong substances that have a sweet taste thanks to the auxiliary ingredients and can look attractive thanks to the use of dyes. They can be used at any time and under any conditions. Many methods of producing lamellas have already been determined, and one of the most commonly used is the solvent-casting method. It owes its popularity to the simplicity of execution and the lack of the need to use special technologies for production. The resulting solution or suspension, with the appropriate composition, is poured into Petri dishes or appropriate molds and then left to dry. Larger films are created, from which smaller ones of precisely defined dimensions are cut out and packed into protective films [7].

The composition of polymer gel films, apart from the drug and the polymer, includes plasticizers, taste masking agents, dyes, and surfactants [13,15]. The most important role in the production of films is played by polymers, which ensure the flexibility of the resulting gel films and allow them to disintegrate when placed in the oral cavity. The polymers selected for the production of films must be thoroughly tested. The most frequently chosen polymers are HPMC (Hydroxypropyl Methyl Cellulose) and HPC (Hydroxypropyl Cellulose). They show good film-forming properties and are widely used in the pharmaceutical industry as polymers for coating tablets, granules, or pellets [8,9,11,12,13,14,15].

The aim of the presented research was to prepare polymer gel films/membranes using the solvent-casting technique, which could be used for orodispersible drug delivery. The following polymers were used: sodium alginate or hydroxypropyl methylcellulose, and the effect of microcrystalline cellulose (mcc) and kollidon (koll), i.e., so-called disintegrators, on the rate of disintegration of the tested films were also investigated [16,17,18,19].

**Figure 1:**
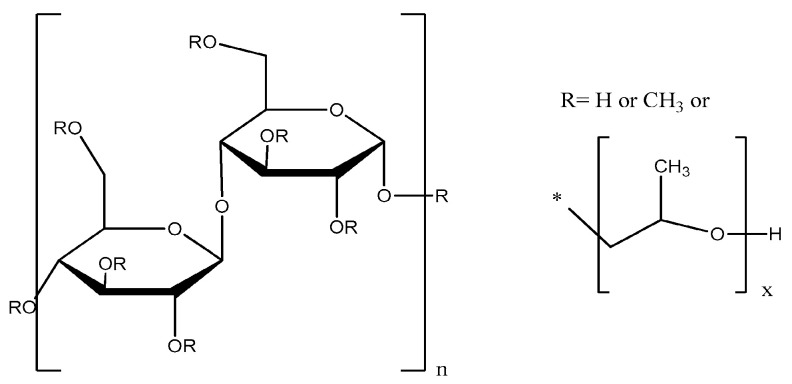
Structure of hypromelose. * point of attachement of the residue to the molecule.

HPMC, otherwise known as hypromellose, belongs to cellulose derivatives (Figure 1). Cellulose is one of the common, natural polymers, which is a linear polysaccharide built of D-glucopyranose units linked by 1,4-β glycosidic bonds. HPMC is a cellulose ether, which is created by replacing the hydroxyl groups of cellulose with methyl and hydroxypropyl groups [16,17].

HPMC dissolves in cold water; when heated to 50–80 °C, it forms a thermally reversible, hard gel. It is insoluble in ethanol and other organic solvents. A pure HPMC solution without additives has a pH of approximately 6.47–7.87 [17].

**Figure 2:**
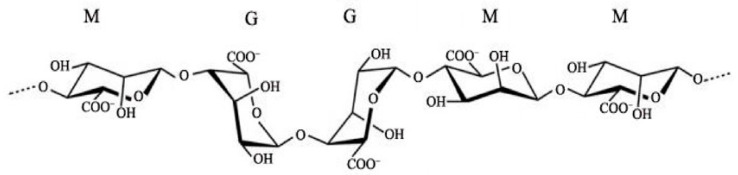
Structure of sodium alginate [19].

Sodium alginate is a linear copolymer of β-D-mannuronic acid (M) residues linked with α-L-guluronic acid (G) with 1,4-glycosidic bonds (Figure 2).

The number of M and G blocks, as well as their distribution, affects the physicochemical properties of alginate. An abundance of M blocks provides a flexible structure and biocompatibility, while a greater amount of G blocks shows a stiffer structure. Alginate has hydroxyl and carboxyl groups located along the skeleton, which can crosslink to form hydrogels [18,19]. It forms a viscous, colloidal solution in water. It is insoluble in chloroform, ether, and in aqueous solution below pH = 3. This compound has excellent filming properties [18].

**Figure 3:**
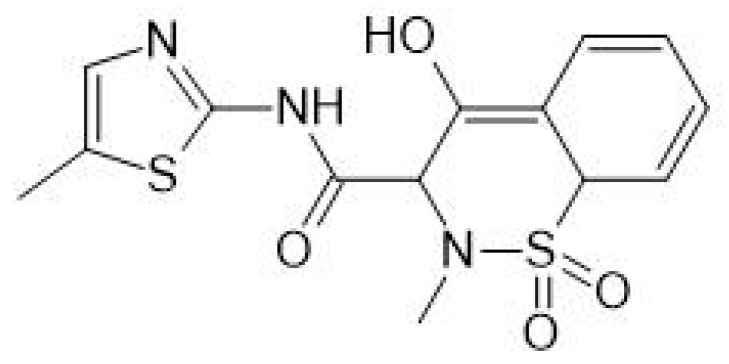
Structure of the meloxicam.

The drug substance selected for the study was meloxicam (Figure 3), a cyclooxygenase inhibitor (COX-2-induced) with analgesic, anti-inflammatory, and antipyretic properties, mainly used in the course of rheumatic diseases in adults and children [20,21,22,23]. It is a drug from the group of non-steroidal anti-inflammatory drugs (NSAIDs) belonging to oxicams, classified as class II BCS (Biopharmaceutical Classification System), which means that it is characterized by low solubility and good permeability, the main characteristics are shown in Table 1.

The evaluation of prepared gel films was based on appearance description, mass uniformity measurement, disintegration time, API content, film wettability, and water content. The dissolution of MLX from prepared gel films was carried out in a basket apparatus using artificial salvia (pH = 6.8). Additionally, API release profiles were analyzed for similarity factors (f_2_) with DDSolver software.

## 2. Results and Discussion

Polymer gel films are one of the formulations that are not as popular as tablets or liquid drugs. This is a form of administering medicinal substances that has been in existence for a long time in pharmacy. Thanks to its properties, it facilitates the use of the substance by people with swallowing problems, people after surgery of the upper digestive tract, children, or the elderly [2,3,6]. The aim of our study was to obtain polymer gel films with the active substance meloxicam and polymers like sodium alginate or hypromellose, and to use disintegrators, microcrystalline cellulose or kollidon, that could be used for orodispersible drug delivery. Four series with different formulations were received, and the ingredients used are presented in Table 2. Then, the differences in the appearance of each series of films, MLX content, water content, and disintegration time were examined, and the water absorption capacity of each series was determined. In addition, the release of MLX from gel films was conducted, and drug substance release profiles were compared for each tested batch.

### 2.1. Appearance of Gel Films

**Figure 4:**
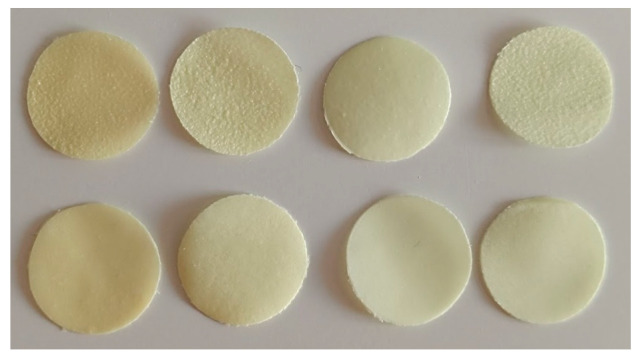
Appearance of prepared gel films.

The appearance of each series of the obtained polymer films is presented in Figure 4. The films in the figure, similarly from the left, are the series: ALG-mcc, ALG-koll, HPMC-mcc, and HPMC-koll.

The upper surface of the film is shown at the top, and the lower surface is at the bottom. The shape of each film in the series is circular. The diameter is always 19 mm because the films were cut using the same suitable mold. The color of each series is yellow due to the color of the active substance, meloxicam; thus, one can suspect a homogeneous distribution of the API over the entire surface of the films. The bottom surface of each film is smooth. The alginate series has a rough top surface, which may have been caused by solvent evaporation. Furthermore, drug–polymer interaction can also cause a rough surface on the films [24]. Comparing the series with hypromellose, only the series with microcrystalline cellulose has a smooth upper surface. When examining the flexibility of the films, films with sodium alginate, which show no traces of bending, are characterized by greater resistance to bending. Both series are resistant to cracking after the strong bending of the film. The discussed observations are summarized in Table 3.

### 2.2. Mass Uniformity of the Gel Films

The highest average film mass was observed in the HPMC-mcc series, 0.1179 g ± 0.011. Subsequently, for the ALG-koll series, the average weight was 0.108 g ± 0.033. The ALG-mcc series weighed an average of 0.096 g ± 0.030. The smallest average film weight was 0.095 g ± 0.020 for the HPMC-koll series. The highest % RSD occurred in the ALG-mcc series, 32%, and was 1% higher than the ALG-koll series. The smallest dispersion of results of 10% characterizes the HPMC-mcc series. The discussed results are presented in Table 4.

### 2.3. Determination of Loading of Meloxicam in Films

The content of the active substance in the obtained polymer films was determined using a validated UV-VIS spectrophotometric method. Four batches of prepared films taken from three different sites of the larger film were evaluated to determine the uniformity of the MLX content.

The results are presented in Table 4. The smallest scutter of results from the mean value occurred in the series ALG-koll and ALG-mcc. RSD% showed the highest values for the HPMC-koll series. This indicates a possible large variation in the content of the API for individual batches of films in this series. The standard deviation for each series takes low values in the range of 0.4–1.3. The content determined experimentally for the ALG-mcc series exceeds the theoretically determined API content for a given average weight by 0.23 mg. This may be the reason for the unevenly dispersed substance in the polymer film. The HPMC-mcc series shows an MLX content of 0.45 mg less than the theoretical. For the ALG-koll series, the average content is 1.1 mg less than the theoretical one. For HPMC-koll, this is the smallest difference of 0.35 mg compared to the theoretical value.

### 2.4. Water Content

**Figure 5:**
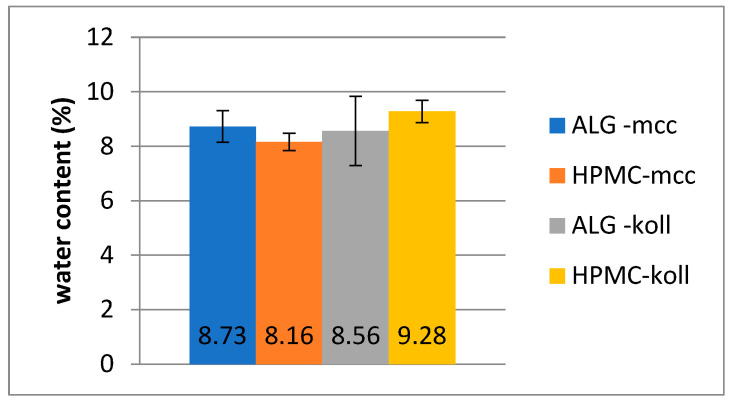
Comparison of water content in prepared films.

The amount of moisture has a significant impact on the mechanical strength and adhesive properties of polymer films [25]. The highest average water content was found in films from the HPMC-koll series, which was 9.28% ± 0.41. For the above series, the RSD% of results is also the largest, 15%. Gel films from the HPMC-mcc series contain the least amount of water, 8.16% ± 0.32 and RSD-4%. The results are presented in Table 4 and illustrated in Figure 5.

In general, it can be observed that the gel films prepared from sodium alginate were characterized by a similar average moisture content (approx. 8%), regardless of the disintegration accelerator used. The water content, as well as water uptake, could influence the condition of the drying but also the properties of the polymer used [25]. Additionally, these properties could be important in the field of mechanical properties like elasticity and elongation.

### 2.5. Disintegration Time

The complete dissolution of each of the films in the system took more than 5 min. The fastest decay was observed for the HPMC-koll series (Table 4), and it averaged 74 s ± 14. This series is also characterized by the smallest RSD of 19%. The longest disintegration time was also observed for the series with hypromellose but with the use of a different disintegrator: microcrystalline cellulose. The HPMC-mcc series film disintegrated into smaller pieces after 160 s ± 62 at an RSD of 39%. The greatest dispersion of results, equal to 72%, characterized the ALG-koll series, whose average decay time was the shortest and amounted to 69 s ± 49. The ALG-mcc series decayed after 107 s ± 40, and its RSD was 38%. The disintegration of each series of films was longer than 30 s but not greater than 180 s, which is the maximum disintegration time for orally disintegrating dosage forms according to the FDA and the US Pharmacopoeia. We also observed that water content in gel film did not influence the disintegration time; all prepared films contained quite a similar amount of water, but different disintegration times were received. This confirms that the speed of disintegration depends on the type of polymer used.

### 2.6. Water Uptake

**Figure 6:**
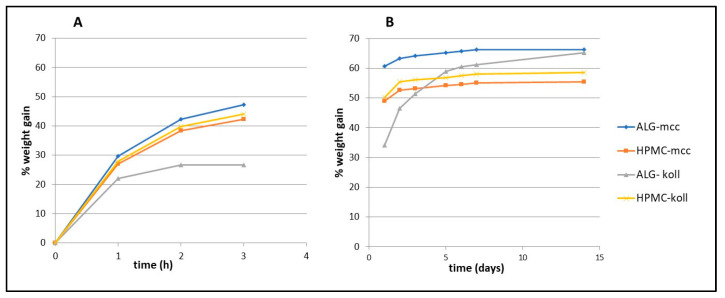
Uptake of water of films: (**A**) growth of mass of films during first 3 h; (**B**) growth of mass during 14 days.

The wettability of the obtained polymer films was tested by placing test tubes with films in a climatic chamber with an air humidity of 80% and a temperature of 25 °C. The change in weight was recorded after a certain time, and the mean weight, standard deviation, and coefficient of variation were calculated for each series; the results are presented in Figure 6. The test was conducted until no noticeable change in weight was found for most of the series, i.e., 14 days.

The ability to absorb moisture plays a key role in controlling drug release and is an essential step required for bioadhesion. The wettability of films is a feature generally observed due to the fact that the polymers selected for the production of films are hydrophilic [5,6]. It was noticed that during the first 3 h of the test, the mass of films from the ALG-mcc series increased the most and the ALG-koll series the least, as seen in Figure 6. After 5 days of testing, the weight of all batches except ALG-koll stabilized. The ALG-koll series showed the greatest differences in weight changes after 5 days. From day 5 of the study, it was noted that the changes for each series were small except for the ALG-koll one.

### 2.7. Dissolution of MLX from Gel Films

**Figure 7:**
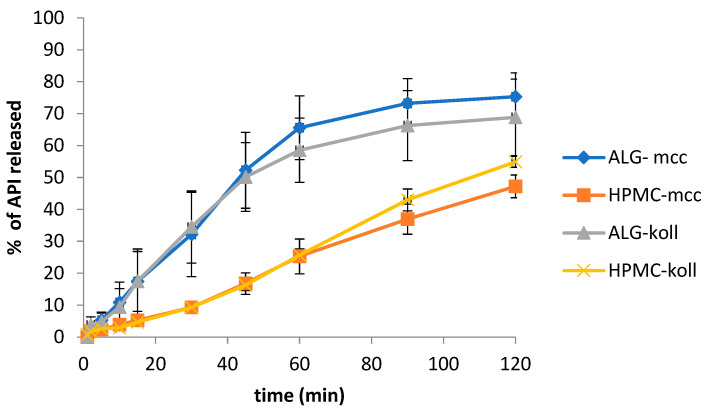
Comparison of MLX release profiles.

For each batch, the study of the dissolution of MLX from polymer gel films in the medium of artificial saliva with a pH of 6.8 was prepared. The study was carried out in a Perlan basket apparatus. All MLX dissolution profiles for each series were compared. The obtained values are summarized in Figure 7. In contact with biological membranes, the polymer film swells by loosening the polymer chain, and API diffusion takes place; the release is directly related to the polymer structure [6,11,15].

Comparing the average amounts of released API from the obtained polymer films after 15 min of testing, the highest amount of meloxicam was released from series ALG-koll at 17.4 ± 9.4%, slightly less than from series ALG-mcc at 17.4 ± 10.2%. The values of released MLX from the remaining batches were much lower, and for HPMC-mcc were 5.2 ± 0.6%, and for HPMC-koll, 4.7 ± 0.4%. After 30 min, the most MLX was also released from series ALG-koll 34.5 ± 11.3% and then from series ALG-mcc 32.2 ± 13.2%. The HPMC-mcc and HPMC-koll series released very similar amounts and showed 9.3 ± 1.7 and 9.3 ± 0.7%, respectively. After 45 min of testing, the largest average amount of released API changed to the ALG-mcc series and amounted to 52.2 ± 11.9%, and from the ALG-koll series, after 45 min, less API was released by 2.1%. The HPMC series showed lower but much similar values. After 2 h of testing, the ALG-mcc series released 75.3 ± 7.5%, ALG-koll 68.8 ± 12.0%, HPMC-koll 54.9 ± 1.7%, and HPMC-mcc 47.2 ± 3.6%. Contrary results were shown by Song et al. [26], who investigated fast-dissolving sublingual films with suspended MLX and also nanoprecipitated MLX. They received the release of almost 40% of MLX during 10 min of the investigation for the suspended MLX and approximately 90% of the release of API from films prepared with nanoprecipitated MLX. Additionally, Sheikh et al. [27], who prepared HPMC orodispersible films with MLX, received almost 85% of API released during the first 5 min of the study, but they used a methanolic solution of MLX during the formulation of gel film. Comparing all release profiles received during our study (Figure 7), the highest average amounts of MLX released into the medium showed gel films consisting of sodium alginate. Significantly less substance was released when the hypromellose polymer was used. Comparing the same substances disintegrating kollidon or microcrystalline cellulose with different polymers show a significant difference in the amount of released MLX. Between the series of ALG-koll and HPMC-koll, the largest difference in the amount of substance released was observed after 45 min of the study and showed that 33.9% more MLX was released from ALG-koll. After 2 min of the study, a difference of 2.1% was shown between the above series. During 60 min of testing, the ALG-mcc series released 40.3% more substances than the HPMC-mcc series, while after the first 2 min, the difference between the sets was only 0.2%. The release profiles were also compared with the composition of the film for the same polymer, sodium alginate or hypromellose, and the use of different microcrystalline cellulose/kollidon disintegrants. It was calculated that in the case of sodium alginate, 1.8% more was released from the series with microcrystalline cellulose (mcc) during the first 2 min of testing and more from the series with kollidon (koll) after 5 and 10 min. The differences were 0.5% and 1.4%, respectively. After 15 min of the study, the difference between the series was only 0.03%. The largest value of the difference observed between the series with alginate was 7.0% more for mcc after 60 min of the study. Comparing the series with hypromellose, it was noticed that they are more similar to each other. During the first 2 min of the test, 0.13% more API was released from the system with mcc. After 5 min, 0.2% more was released from the system with kollidon. No difference in the release occurred after 30 min of the test, and the largest difference was observed at the endpoint (120 min) of the test, showing that the kollidon series released 8.0% more than the microcrystalline cellulose series. However, these results confirm that the received gel films did not act as fast-dissolving drug delivery systems.

### 2.8. Comparison of Release Profiles with DDSolver Software

The received release profiles were analyzed by DDSolver software [28], and the results of the comparison of the MLX release/dissolution profiles from the obtained polymer gel films using the f_2_ similarity coefficients are presented in Table 5.

Analyzing the results presented in the table above, it can be seen that the similarity [28,29] of the profiles (f_2_ > 50) occurs between the HPMC-mcc and HPMC-koll series; it is the highest and amounts to 74.21. There is also a similarity of 69.53 between the ALG-mcc and ALG-koll series. This means the disintegrant does not influence the dissolution of MLX. Additionally, similarity was not observed using the same disintegrant and different polymers. Other series comparisons also show no similarity (f_2_ < 50). This analysis showed that the type of polymer used has a greater influence on the similarity of release profiles than the type of disintegrants used.

## 3. Conclusions and Perspectives

Analyzing the results obtained during the study it can be concluded that the use of the solvent casting method allowed us to obtain polymer gel films with a similar appearance, regardless of the polymer or disintegrant used. This method makes it really easy to prepare films with good quality. The produced series of films contain a similar value of MLX in relation to the value calculated theoretically, so it could be possible to prepare the film with the specified content of API. The developed method of obtaining films allowed us to obtain gel films with water content below 10%, and the content of water did not influence the water uptake. Unfortunately, the disintegration time of the films of each series was longer than 30 s and did not meet the assumptions of the disintegration time of “quickly disintegrating” dosage forms. Although, for a given polymer, the release profiles of the active substance were similar regardless of the disintegrant used; for Oro-dispersible forms of drugs, some changes in the composition are required to make the disintegration faster. The summarized proposed composition shows that polymers like sodium alginate or hypromellose, which are widely used as the excipients, have big potential as film-forming agents. Additionally, some similarity of action for disintegrants cellulose and kollidone was proven. Two different excipients did not influence the release of API while influencing the speed of disintegration time. However, because we failed with the fast disintegration, the proposed formulations require future development. There are a lot of excipients which influence the speed of disintegration [30,31], called super disintegrants. Future investigation assumes some changes in the proportions between polymer and disintegrant and also the use of some other examples of disintegrants like crosslinked cellulose, sodium starch glycolate, or crosslinked starch [32,33]. All mentioned substances work well as disintegrants for tablets; therefore, it seems they also have a big potential for orodispersible films.

## 4. Materials and Methods

### 4.1. Materials

The model drug Meloxicam (MLX) was a gift from Biofarm (Poznan, Poland) and was used as received. Sodium alginate was purchased from Sigma-Aldrich, HPMC was purchased from Colorcon Limited, Kollidon CL from BASF ChemTrade GmbH, and MCC-Emocel 90XLM was purchased from JRS Pharma. Sodium hydroxide, sodium chloride, and kalium dihydrophosphate were purchased from Avantor Performance Materials S.A. Disodium bicarbonate and phosphate acid were received from Chempur.

### 4.2. Preparation of Polymer Gel Films

Polymer films were prepared by the solvent casting method [4,7]. Four different series of gel films were prepared with the same content of the active substance (MLX), glycerol (plasticizer) and aspartame (sweetening agent). The polymers used were hydroxypropyl methylcellulose (HPMC) and sodium alginate (ALG). Films also contained microcrystalline cellulose or kollidon as disintegrating agents. The weight of each ingredient is listed in Table 2 (it is the amount used per 100 g of aqueous solution which was then poured into 4 Petri dishes). Preparation of the films was started with the preparation of polymeric solutions that were used to suspend the other ingredients. For films based on sodium alginate, 3 g of sodium alginate were dissolved in 60 g of water using a magnetic stirrer at 600 rpm and a temperature of 60 °C. After 45 min of mixing, microcrystalline cellulose (mcc) or kollidon (Koll) was added to the alginate solution, depending on the prepared batch, and further stirred on a magnetic stirrer. Then, meloxicam and glycerol were mixed in an evaporating dish and added to the previously prepared suspension. The whole was stirred for approximately 30 min to reach a temperature of roughly 37 °C. Aspartame was dissolved in 30 g of water and added to the cooled solution. The whole was stirred for another 5 min. For films with HPMC, 20 g of water were weighed and heated to 60 °C. Then, 3 g of HPMC were added and mixed for 5 min. Subsequently, 40 g of cold water were added and stirred for 30 min with the help of a magnetic stirrer using 600 rpm and a temperature of 50 °C. After this time, MCC or Koll, depending on the batch being prepared, was added, and mixing was continued for 10 min. Meloxicam and glycerol were mixed in an evaporating dish and added to the mixing ingredients. The whole was stirred until the temperature reached 37 °C to add aspartame dissolved in 30 g of water. The whole was stirred for another 5 min. For each series, 25 g of the prepared gel film-forming mass were poured onto 4 Petri dishes. The dishes were placed in an oven for 48 h at 40 °C. After the time had elapsed, the plates were removed from the oven, and smaller films, 1.9 cm in diameter, were cut out from each large film, approximately 10 cm in diameter, as shown in Figure 4. The obtained films were packed in aluminum foil and stored in a desiccator for subsequent examinations.

### 4.3. Characterization of Prepared Gel Films

The obtained polymer gel films with MLX were tested in order to determine their properties. The appearance and size of the films, their weight, moisture content, meloxicam content, and disintegration time of the films were determined. Wettability in the climatic chamber was also evaluated, and the dissolution of MLX from the obtained films was evaluated.

#### 4.3.1. Appearance, Size, and Mass Uniformity of the Gel Films

The obtained films were evaluated, taking into account their shape, dimensions, surface, color, and flexibility. Shape was defined and it was checked whether each film had the same shape. For 10 randomly cut films from a given series, the diameter was measured with an accuracy of 1 mm. The surface was checked to determine whether it was smooth or rough or if there was a visible deposit or traces of cracks. Color was checked to determine whether it was uniform for each film, without a visible deposit of the active substance in one place. Flexibility was checked to determine whether the films had cracks/disintegrated after bending. The randomly selected films of each batch were weighed on an analytical balance, and the mean weight, standard deviation, and coefficient of variation for each batch were calculated.

#### 4.3.2. Water Content

The reference method for determining the water content of many substances is Karl Fischer titration. It is effective in determining the water content from a few ppm to very high values close to 100%. The analysis consists of the oxidation of sulfur dioxide with a methanolic hydroxide solution. Determination of water in the tested polymer gel films was performed using the Karl Fisher Mettler Toledo titrator. The measurement was carried out using the volumetric method. The mentioned method was developed by the system of the device. The analysis was carried out using Titrant 5, a two-component reagent for Karl Fisher titration Aquastar, Merck, and a solvent of the mentioned company. The measurement was started by filling the titration cell with solvent to a volume of 40 mL. Then, after selecting the previously developed method, the apparatus was prepared for determination. After the instrument was brought to anhydrous conditions by adding a titrant to a constant drift, the instrument was ready for analysis. The polymer film was weighed and placed in the titration cell. After 5 min of mixing the sample in the solvent with a magnetic stirrer, the system of the device automatically began the determination of water released from the sample. For each series of films, the determination was repeated 3 times.

#### 4.3.3. Content of MLX in Gel Films

**Figure 8:**
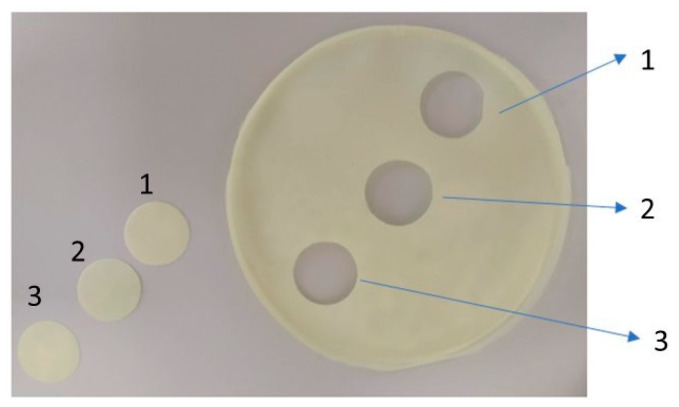
Explanation of the content of MLX in gel films. (Numbers 1–3 determine the places of cut of small films, also marked in the figure).

The determination of the amount of MLX in each series of gel films was conducted for each series of films at 3 different points, as marked in Figure 8.

Thanks to this, it was possible to determine whether the pouring method guarantees a similar content of meloxicam in the films cut from each place in the Petri dish. The films were weighed, and each was individually allowed to dissolve in 50 mL 0.1 M NaOH for 24 h. After a predetermined time, the dissolved film solutions were filtered through 0.22 µm membrane filters. In total, 1.5 mL of each filtered solution was extracted and made up to 20 mL with 0.1 M NaOH. In the samples prepared in this way, the content of meloxicam was determined using a validated UV-Vis spectrophotometric method. The absorbance was measured at a wavelength of 361 nm. The amount of MLX contained in the film was expressed as a percentage, and calculations were carried out using the equation of the 0.1 M NaOH standard curve.

#### 4.3.4. Disintegration Time of Gel Films

The study of disintegration time was carried out by placing the film in the trail with 10 mL of artificial saliva, pH 6.8, and a temperature of 37 °C. The study was performed as described by M. Scarpa et al. [24] with slight modification. In the plate, the film was stirred by a 4 cm long magnetic stirrer, which moved at a speed of 100 rpm to imitate the movements of the tongue in the mouth.

**Figure 9:**
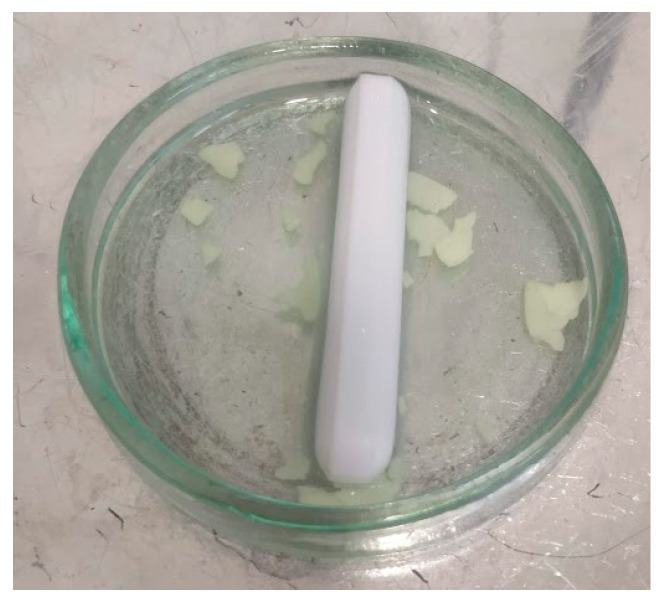
Endpoint of disintegration time.

Figure 9 shows the moment considered to be the time of film disintegration (breaking into smaller parts).

Films in an area of 2–8 cm^2^ should usually disintegrate within 30 s. However, there are no clear pharmacopoeial requirements for a maximum acceptable disintegration time for films. For these reasons, the requirements of the FDA and US Pharmacopoeia guidelines for orally disintegrating tablets (ODT) are often used for polymer films. The maximum disintegration time for ODT is ≤3 min [1].

#### 4.3.5. Wettability of the Films

The wettability test was carried out in the Jeio Tech climatic chamber at 80% air humidity and 25 °C temperature. Measurements were collected for each series, from which 3 random films were selected, weighed, and placed in plastic, closed tubes. After reaching the test conditions in the climatic chamber, closed tubes were inserted into it and opened with the chamber closed by means of access through sluices. On the first day of the study, the samples were weighed 3 times every hour and then every several hours. An Ohaus analytical balance with an accuracy of 0.0001 g was used to determine changes in weight. The study was terminated with no weight changes observed after 14 days.

#### 4.3.6. Dissolution Study

The release of MLX from the obtained polymer films was carried out using a basket apparatus (Perlan) [4,6]. Previously weighed films with the expected content of 5 mg of MLX per 0.1 g of gel film mass were placed in each basket. The test was carried out in 3 repetitions for each series of films in the medium of artificial saliva (pH = 6.8), heated to the temperature of 37 ± 0.5 °C. The volume of the release fluid was 900 mL. The test time was 120 min. The baskets rotated at 100 rpm. Then, 4 mL samples were automatically extracted to the Perlan sample storage station at 1, 2, 5, 10, 15, 30, 45, 60, 90, and 120 min. Experiment: The artificial saliva volume was automatically replenished with fresh release solution each time. MLX content was determined in each sample using a validated UV-Vis spectrophotometric method. The amount of meloxicam released was expressed as a percentage using the following Equation (1), where U% is the amount of released MLX (%); M_t_ is the mass of MLX in 900 mL of dissolution medium during time t (mg); ∑M_previous_ is the sum of the content of MLX in previous samples (mg); X_declared_ is the expected content of MLX.

(1) $$U\%=\frac{(M_{t}+\sum M_{previous})\times 100}{X_{declared}}.$$

#### 4.3.7. Comparison of Release Profiles for the Similarities

The obtained meloxicam release profiles from the prepared polymer gel films were used to assess their similarity using similarity factor f_2_ [28,29]. The statistical method, independent of the model used for comparison, was established using the DDSolver program. The following equation [26] was used in the calculations (2), where f_2_ is the similarity coefficient, R_t_; T_t_ is the % dissolved/released API for the evaluated sample and reference sample in timepoint t; n is the number of samples; t is the timepoint.

(2) $$f_{2}=50\times \log\{{[1+\frac{1}{n}\sum_{i=1}^{n}{\left(R_{t}-T_{t}\right)}^{2}]}^{-0.5}\times 100\}.$$

## Figures and Tables

**Table 1:** Physico-chemical characteristics of meloxicam.

Properties	Meloxicam			Publ.
**state**	yellow solid state			[20]
**molecular mass (g/mol)**	351.401			[21]
**BCS class**	II			[22]
**melting temperature (°C)**	255			
**solubility** **(mg/mL)**	water		0.012	[22,23]
	glycerol		0.138	
	NaOH	pH = 7.4	0.062	
		pH = 9.6	2.615	
		pH = 10.7	17.900	
	Ethanol		0.354	
	PEG-400		3.763	
**pKa**	pH 0–3		1.09	[23]
	pH 2.5–6.5		4.18	
**logP**	pH = 2		2.43	
	pH = 4		2.34	
	pH = 6		1.01	
	pH = 7		0.07	

**Table 2:** Description of the main components.

Ingredient *	ALG-mcc	ALG-coll	HPMC-mcc	HPMC-koll
	Weight (g)			
**sodium alginate**	3.0	3.0	-	-
**HPMC**	-		3.0	3.0
**kollidon**	-	2.0	-	2.0
**microcrystalline cellulose**	2.0	-	2.0	-
**meloxicam**	0.5	0.5	0.5	0.5
**glicerol**	5.0	5.0	5.0	5.0
**aspartame**	0.275	0.275	0.275	0.275

* Composition for 100 g of gel film mass.

**Table 3:** Summary of film appearance.

Series	ALG-mcc	ALG-koll	HPMC-mcc	HPMC-koll
Shape	Round			
Diameter	19 mm			
Surface of upper side	Rough	Rough	smooth	Rough
Surface of lower side	smooth			
Color	Yellow			
Elasticity	High elasticity Does not break when bent No trace when bend is visible		Low elasticity Does not break when bent There is a trace of the fold	

**Table 4:** Characteristics of gel films.

Property		Series			
		ALG-mcc	HPMC-mcc	ALG-koll	HPMC-koll
**Mass (g)**	X_śr_ ± SD	0.096 ± 0.030	0.117 ± 0.011	0.108 ± 0.033	0.095 ± 0.020
**Content of MLX** **(mg)**	Determined X_śr_ ± SD	4.85 ± 0.40	5.53 ± 0.51	4.11 ± 0.34	5.16 ± 1.29
	theoretical	4.62	5.98	5.21	5.51
**Water content (%)**	X_śr_ ± SD	8.73 ± 0.58	8.16 ± 0.32	8.56 ± 1.27	9.28 ± 0.41
**Disintegration time (s)**	X_śr_ ± SD	107 ± 40	160 ± 62	69 ± 49	74 ± 14

**Table 5:** Similarity coefficients f_2_ of release profiles of MLX.

Comparing Series	Similarity Coefficient f_2_		
	HPMC-mcc	ALG-koll	HPMC-koll
**ALG-mcc**	31.05	69.53 *	32.55
**HPMC-mcc**		33.97	74.21 *
**ALG-koll**			35.43

* similar release profiles.

## Data Availability

Not applicable.
